# Supplementary material for: Polyketide synthase gene clusters in the ericoid mycorrhizal fungus Oidiodendron maius: genome-wide prediction, expression profiling, and functional characterization of knockout mutants
Source: Front Plant Sci. 2026 Sep 16;17:1924430. doi: 10.3389/fpls.2026.1924430 (PMC13623903; doi:10.3389/fpls.2026.1924430)
Supplement: Supplementary file 1 [file SupplementaryFile1.zip › Supplementary Materials/Supplementary Folder 1.zip!/Oidma1_AssemblyScaffolds_Repeatmasked/knownclusterblast/cluster16/jgi.p_Oidma1_119691_mibig_hits.html]

| MIBiG Protein | Description | MIBiG Cluster | MiBiG Product | % ID | % Coverage | BLAST Score | E-value |
| --- | --- | --- | --- | --- | --- | --- | --- |
| ACY02004.1 | phospho-2-dehydro-3-deoxyheptonate\_aldolase | BGC0000343 | Enterobactin\_biosynthetic\_gene\_cluster | 45 | 87.0558375635 | 289 | 4.8e-78 |
| AAO07756.1 | 3-deoxy-7-phosphoheptulonate\_synthase | BGC0000460 | Vulnibactin\_biosynthetic\_gene\_cluster | 46 | 83.5025380711 | 276 | 5.4e-74 |
| AJI44169.1 | 2-keto-3-deoxy-D-arabino-heptulosonate-7 | BGC0001193 | Thiolutin\_biosynthetic\_gene\_cluster | 44 | 87.8172588832 | 270 | 3.9e-72 |
| AKP45401.1 | CysN | BGC0001413 | Cystobactamide\_biosynthetic\_gene\_cluster | 44 | 82.9949238579 | 266 | 4.3e-71 |
| CAJ45633.1 | DAHP\_synthetase | BGC0000454 | Vanchrobactin\_biosynthetic\_gene\_cluster | 46 | 77.9187817259 | 248 | 1.6e-65 |
| ACU36652.1 | phospho-2-dehydro-3-deoxyheptonate\_aldolase | BGC0000392 | Mirubactin\_biosynthetic\_gene\_cluster | 39 | 87.0558375635 | 234 | 1.8e-61 |
